# Diaqua­bis­[2-(5-isopropyl-5-methyl-4-oxo-4,5-dihydro-1*H*-imidazol-2-yl-κ*N* ^3^)nicotinato-κ*N*]manganese(II)

**DOI:** 10.1107/S1600536810048506

**Published:** 2010-11-27

**Authors:** Peng Gao, Ji-Zhong Liu, Zhong Zhang, Zhong-Jing Huang

**Affiliations:** a Department of Chemistry, Guangxi University for Nationalities, Nanning 530006, People’s Republic of China

## Abstract

In the title compound, [Mn(C_13_H_14_N_3_O_3_)_2_(H_2_O)_2_], the Mn^II^ ion is coordinated by four N atoms from two (±)-2-(5-isopropyl-5-methyl-4-oxo-4,5-dihydro-1*H*-imidazol-2-yl)nicotinate ligands and two water mol­ecules in a distorted octa­hedral environment. Inter­molecular O—H⋯O hydrogen bonds lead to a chain along [010]. Intra­molecular N—H⋯O and O—H⋯O hydrogen bonds are observed.

## Related literature

For coordination compounds with pyridine­carb­oxy­lic acids, see: Chatterjee *et al.* (1998 ▶); Nathan & Mai (2000 ▶); Park *et al.* (2007 ▶); Yang *et al.* (2002 ▶). For the synthesis of compounds containing imidazolidinone derivatives, see: Erre *et al.* (1998 ▶).
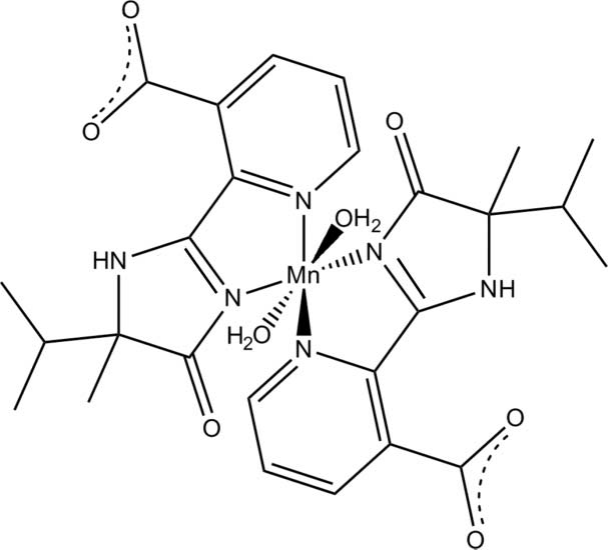


## Experimental

### 

#### Crystal data

[Mn(C_13_H_14_N_3_O_3_)_2_(H_2_O)_2_]
*M* *_r_* = 611.52
Orthorhombic, 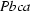

*a* = 12.620 (3) Å
*b* = 19.753 (4) Å
*c* = 23.017 (5) Å
*V* = 5738 (2) Å^3^
*Z* = 8
Mo *K*α radiation
μ = 0.52 mm^−1^
*T* = 298 K
0.50 × 0.48 × 0.35 mm

#### Data collection

Bruker SMART 1000 diffractometer
Absorption correction: multi-scan (*SADABS*; Sheldrick, 1996 ▶) *T*_min_ = 0.782, *T*_max_ = 0.839
25491 measured reflections
5057 independent reflections
3208 reflections with *I* > 2σ(*I*)
*R*_int_ = 0.055

#### Refinement

*R*[*F* ^2^ > 2σ(*F* ^2^)] = 0.057
*wR*(*F* ^2^) = 0.179
*S* = 1.06
5057 reflections
376 parameters
5 restraints
H-atom parameters constrained
Δρ_max_ = 1.08 e Å^−3^
Δρ_min_ = −0.47 e Å^−3^

### 

Data collection: *SMART* (Bruker, 2007 ▶); cell refinement: *SAINT* (Bruker, 2007 ▶); data reduction: *SAINT*; program(s) used to solve structure: *SHELXS97* (Sheldrick, 2008 ▶); program(s) used to refine structure: *SHELXL97* (Sheldrick, 2008 ▶); molecular graphics: *DIAMOND* (Brandenburg, 1999 ▶); software used to prepare material for publication: *SHELXTL* (Sheldrick, 2008 ▶).

## Supplementary Material

Crystal structure: contains datablocks I, global. DOI: 10.1107/S1600536810048506/hy2382sup1.cif

Structure factors: contains datablocks I. DOI: 10.1107/S1600536810048506/hy2382Isup2.hkl

Additional supplementary materials:  crystallographic information; 3D view; checkCIF report

## Figures and Tables

**Table 1:** Hydrogen-bond geometry (Å, °)

*D*—H⋯*A*	*D*—H	H⋯*A*	*D*⋯*A*	*D*—H⋯*A*
N2—H2⋯O2	0.86	1.74	2.524 (5)	151
N5—H5⋯O5	0.86	1.76	2.535 (6)	149
O7—H7*A*⋯O3	0.85	2.09	2.838 (5)	147
O7—H7*B*⋯O1^i^	0.85	1.80	2.638 (5)	170
O8—H8*A*⋯O6	0.85	2.06	2.791 (5)	143
O8—H8*B*⋯O4^ii^	0.85	1.77	2.609 (5)	171

Symmetry codes: (i) 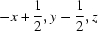; (ii) 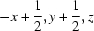.

**Fig. 1.**
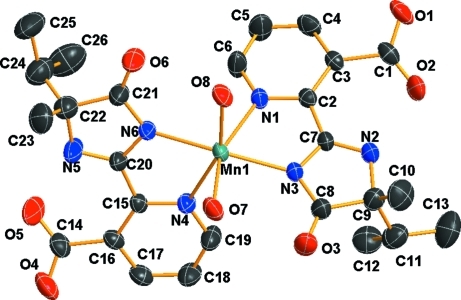
Molecular structure of the title compound. Displacement ellipsoids are drawn at the 50% probability level. H atoms have been omitted for clarity.

**Fig. 2.**
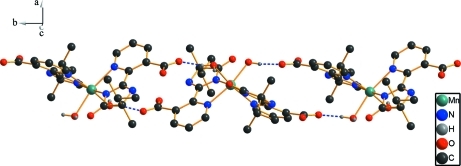
Part of the chain structure in the title compound. Dashed lines indicate hydrongen bonds.

## supplementary crystallographic information

### Comment

(±)-2-(4-Isopropyl-4-methyl-5-oxo-4,5-dihydro-1*H*-imidazol-2-yl) nicotinic acid (imina) is a novel pyridylimidazolidinone ligand, which provides with efficient metal-chelating ability. The pyridine carboxylic acids have been extensively used in the design of coordination compounds, due to a variety of bonding modes and ability to form strong hydrogen bonds (Chatterjee *et al.*, 1998; Nathan & Mai, 2000; Park *et al.*, 2007; Yang *et al.*, 2002). Imidazole group, which is one of the polydentate amine ligands, generally coordinates to metal ions using N atoms as donors. The synthesis of imina and its manganese(II) complex has been reported (Erre *et al.*, 1998). Here we present the structure of a manganese(II) complex with 2-(5-isopropyl-5-methyl-4-oxo-4,5-dihydro-1*H*- imidazol-2-yl)nicotinate (*L*) ligand.

The molecular structure of the title complex is shown in Fig. 1. The asymmetric unit contains one Mn^II^ atom, two *L* ligands and two coordinated water molecules. The Mn^II^ atom exhibits a distorted octahedral geometry, defined by four N atoms from two *L* ligands and two O atoms from two water molecules. The dihedral angle between the two *L* planes in the complex is 61.58 (9)°. Intramolecular N—H···O and O—H···O hydrogen bonds are observed (Table 1). The complex molecules are connected via intermolecular O—H···O hydrogen bonds, forming a one-dimensional chain (Fig. 2).

### Experimental

A mixture of Mn(CH_3_CO_2_)_2_.4H_2_O (0.122 g, 0.5 mmol), imina (0.392 g, 0.5 mmol), DMF (5 ml) and H_2_O (15 ml) was heated in a Teflon-lined steel bomb at 423 K for 3 d. Yellow crystals were obtained by slow evaporation of the solution at room temperature (yield: 78% ). Analysis, calculated for C_26_H_32_MnN_6_O_8_: C 51.07, H 5.27, N 13.74%; found: C 51.02, H 5.23, N 13.70%.

### Refinement

H atoms on C and N atoms were positioned geometrically and refined using a riding model, with C—H = 0.93–0.98 Å and N—H = 0.86 Å and with *U*_iso_(H) = 1.2(1.5 for methyl)*U*_eq_(C,N). The water H atoms were located in a difference Fourier map and refined as riding atoms, with O—H = 0.85 Å and *U*_iso_(H) = 1.2*U*_eq_(O). The highest residual electron density was found 1.08 Å from C22 and the deepest hole 0.33 Å from N5.

### Crystal data

**Table:**

[Mn(C_13_H_14_N_3_O_3_)_2_(H_2_O)_2_]	*F*(000) = 2552
*M**_r_* = 611.52	*D*_x_ = 1.416 Mg m^−^^3^
Orthorhombic, *P**b**c**a*	Mo *K*α radiation, λ = 0.71073 Å
Hall symbol: -P 2ac 2ab	Cell parameters from 5733 reflections
*a* = 12.620 (3) Å	θ = 2.4–27.9°
*b* = 19.753 (4) Å	µ = 0.52 mm^−^^1^
*c* = 23.017 (5) Å	*T* = 298 K
*V* = 5738 (2) Å^3^	Block, yellow
*Z* = 8	0.50 × 0.48 × 0.35 mm

### Data collection

**Table:**

Bruker SMART 1000 diffractometer	5057 independent reflections
Radiation source: fine-focus sealed tube	3208 reflections with *I* > 2σ(*I*)
graphite	*R*_int_ = 0.055
φ and ω scans	θ_max_ = 25.0°, θ_min_ = 1.8°
Absorption correction: multi-scan (*SADABS*; Sheldrick, 1996)	*h* = −14→15
*T*_min_ = 0.782, *T*_max_ = 0.839	*k* = −23→23
25491 measured reflections	*l* = −14→27

### Refinement

**Table:**

Refinement on *F*^2^	Primary atom site location: structure-invariant direct methods
Least-squares matrix: full	Secondary atom site location: difference Fourier map
*R*[*F*^2^ > 2σ(*F*^2^)] = 0.057	Hydrogen site location: inferred from neighbouring sites
*wR*(*F*^2^) = 0.179	H-atom parameters constrained
*S* = 1.06	*w* = 1/[σ^2^(*F*_o_^2^) + (0.0592*P*)^2^ + 17.1897*P*] where *P* = (*F*_o_^2^ + 2*F*_c_^2^)/3
5057 reflections	(Δ/σ)_max_ < 0.001
376 parameters	Δρ_max_ = 1.08 e Å^−^^3^
5 restraints	Δρ_min_ = −0.47 e Å^−^^3^

### Fractional atomic coordinates and isotropic or equivalent isotropic displacement parameters (Å^2^)

**Table:**

	*x*	*y*	*z*	*U*_iso_*/*U*_eq_	
Mn1	0.28498 (5)	0.10909 (3)	0.37476 (3)	0.0311 (2)	
N1	0.1631 (3)	0.19583 (18)	0.39468 (16)	0.0367 (9)	
N2	0.2232 (4)	0.2701 (2)	0.25716 (17)	0.0490 (11)	
H2	0.1965	0.3100	0.2544	0.059*	
N3	0.2672 (3)	0.17162 (18)	0.29797 (15)	0.0355 (9)	
N4	0.1688 (3)	0.02058 (18)	0.35275 (16)	0.0365 (9)	
N5	0.2283 (4)	−0.0537 (2)	0.49061 (18)	0.0664 (15)	
H5	0.2023	−0.0939	0.4929	0.080*	
N6	0.2631 (3)	0.04717 (18)	0.45175 (15)	0.0350 (9)	
O1	0.0913 (3)	0.42772 (17)	0.36537 (17)	0.0651 (11)	
O2	0.1213 (4)	0.37586 (18)	0.28288 (17)	0.0650 (12)	
O3	0.3547 (3)	0.12123 (19)	0.22133 (16)	0.0646 (11)	
O4	0.0995 (3)	−0.21079 (17)	0.38402 (18)	0.0673 (12)	
O5	0.1178 (5)	−0.1567 (2)	0.46503 (19)	0.110 (2)	
O6	0.3470 (3)	0.09912 (18)	0.52887 (15)	0.0637 (11)	
O7	0.4045 (3)	0.05333 (16)	0.32650 (15)	0.0475 (9)	
H7A	0.3963	0.0573	0.2900	0.057*	
H7B	0.4022	0.0115	0.3351	0.057*	
O8	0.3971 (2)	0.16651 (15)	0.42592 (14)	0.0440 (8)	
H8A	0.3819	0.1647	0.4619	0.053*	
H8B	0.3977	0.2080	0.4162	0.053*	
C1	0.1092 (3)	0.3765 (2)	0.3365 (2)	0.0391 (11)	
C2	0.1627 (3)	0.2491 (2)	0.35795 (19)	0.0308 (10)	
C3	0.1138 (3)	0.3108 (2)	0.37203 (19)	0.0301 (10)	
C4	0.0613 (4)	0.3130 (2)	0.4252 (2)	0.0468 (13)	
H4	0.0279	0.3528	0.4366	0.056*	
C5	0.0578 (5)	0.2576 (3)	0.4611 (2)	0.0643 (17)	
H5A	0.0199	0.2587	0.4958	0.077*	
C6	0.1118 (5)	0.2006 (3)	0.4444 (2)	0.0565 (15)	
H6	0.1123	0.1635	0.4693	0.068*	
C7	0.2186 (4)	0.2319 (2)	0.30315 (18)	0.0341 (10)	
C8	0.3063 (4)	0.1685 (2)	0.2425 (2)	0.0461 (12)	
C9	0.2812 (4)	0.2346 (3)	0.2106 (2)	0.0473 (12)	
C10	0.3824 (5)	0.2715 (3)	0.1936 (3)	0.0673 (17)	
H10A	0.3653	0.3162	0.1803	0.101*	
H10B	0.4173	0.2470	0.1631	0.101*	
H10C	0.4284	0.2744	0.2267	0.101*	
C11	0.2073 (5)	0.2211 (3)	0.1594 (2)	0.0609 (15)	
H11	0.2455	0.1906	0.1331	0.073*	
C12	0.1066 (5)	0.1839 (3)	0.1777 (3)	0.0666 (17)	
H12A	0.1252	0.1447	0.2002	0.100*	
H12B	0.0682	0.1700	0.1437	0.100*	
H12C	0.0632	0.2135	0.2006	0.100*	
C13	0.1825 (7)	0.2845 (4)	0.1247 (3)	0.095 (2)	
H13A	0.1352	0.2734	0.0935	0.142*	
H13B	0.2469	0.3029	0.1091	0.142*	
H13C	0.1497	0.3174	0.1497	0.142*	
C14	0.1122 (4)	−0.1588 (2)	0.4119 (2)	0.0452 (12)	
C15	0.1655 (3)	−0.0322 (2)	0.39010 (18)	0.0309 (10)	
C16	0.1191 (3)	−0.0941 (2)	0.3754 (2)	0.0339 (10)	
C17	0.0734 (4)	−0.0981 (2)	0.3206 (2)	0.0443 (12)	
H17	0.0420	−0.1385	0.3088	0.053*	
C18	0.0735 (5)	−0.0442 (3)	0.2836 (2)	0.0543 (14)	
H18	0.0406	−0.0469	0.2475	0.065*	
C19	0.1235 (4)	0.0142 (3)	0.3011 (2)	0.0518 (14)	
H19	0.1256	0.0508	0.2756	0.062*	
C20	0.2189 (4)	−0.0141 (2)	0.44567 (19)	0.0373 (11)	
C21	0.3027 (5)	0.0501 (3)	0.5075 (2)	0.0505 (13)	
C22	0.2893 (5)	−0.0204 (3)	0.5368 (2)	0.0533 (12)	
C23	0.3975 (5)	−0.0538 (3)	0.5459 (3)	0.0712 (17)	
H23A	0.3879	−0.0982	0.5620	0.107*	
H23B	0.4390	−0.0269	0.5721	0.107*	
H23C	0.4336	−0.0573	0.5093	0.107*	
C24	0.2253 (5)	−0.0186 (4)	0.5912 (3)	0.0735 (16)	
H24	0.2100	−0.0655	0.6021	0.088*	
C25	0.2858 (7)	0.0140 (4)	0.6421 (3)	0.101 (3)	
H25A	0.3512	−0.0097	0.6482	0.152*	
H25B	0.2434	0.0115	0.6767	0.152*	
H25C	0.3004	0.0606	0.6332	0.152*	
C26	0.1188 (6)	0.0170 (4)	0.5812 (3)	0.100 (2)	
H26A	0.1310	0.0639	0.5726	0.150*	
H26B	0.0760	0.0132	0.6155	0.150*	
H26C	0.0828	−0.0039	0.5491	0.150*	

### Atomic displacement parameters (Å^2^)

**Table:**

	*U*^11^	*U*^22^	*U*^33^	*U*^12^	*U*^13^	*U*^23^
Mn1	0.0387 (4)	0.0209 (3)	0.0337 (4)	−0.0009 (3)	−0.0012 (3)	0.0038 (3)
N1	0.047 (2)	0.029 (2)	0.034 (2)	0.0061 (17)	0.0065 (18)	0.0057 (17)
N2	0.078 (3)	0.031 (2)	0.037 (2)	0.015 (2)	0.004 (2)	0.0046 (19)
N3	0.049 (2)	0.0263 (19)	0.031 (2)	0.0067 (17)	0.0038 (17)	0.0039 (17)
N4	0.044 (2)	0.032 (2)	0.033 (2)	−0.0081 (17)	−0.0043 (18)	0.0033 (17)
N5	0.115 (4)	0.046 (3)	0.039 (2)	−0.034 (3)	−0.013 (3)	0.012 (2)
N6	0.047 (2)	0.0271 (19)	0.0307 (19)	−0.0084 (17)	−0.0055 (17)	0.0025 (17)
O1	0.097 (3)	0.0228 (18)	0.076 (3)	0.0018 (19)	0.020 (2)	−0.0028 (18)
O2	0.105 (3)	0.038 (2)	0.052 (2)	0.029 (2)	−0.003 (2)	0.0107 (18)
O3	0.097 (3)	0.051 (2)	0.046 (2)	0.028 (2)	0.019 (2)	0.0007 (18)
O4	0.090 (3)	0.0272 (19)	0.085 (3)	−0.0018 (19)	−0.016 (2)	0.003 (2)
O5	0.220 (6)	0.056 (3)	0.052 (3)	−0.075 (3)	−0.003 (3)	0.016 (2)
O6	0.099 (3)	0.050 (2)	0.042 (2)	−0.032 (2)	−0.020 (2)	0.0020 (18)
O7	0.056 (2)	0.0297 (17)	0.056 (2)	0.0084 (16)	0.0059 (18)	0.0029 (16)
O8	0.052 (2)	0.0286 (17)	0.052 (2)	−0.0086 (15)	−0.0028 (17)	0.0019 (16)
C1	0.031 (2)	0.027 (3)	0.059 (3)	0.0029 (19)	−0.002 (2)	−0.001 (2)
C2	0.030 (2)	0.028 (2)	0.034 (2)	0.0033 (18)	−0.0049 (19)	0.0007 (19)
C3	0.025 (2)	0.026 (2)	0.039 (2)	0.0009 (17)	−0.005 (2)	−0.001 (2)
C4	0.048 (3)	0.036 (3)	0.056 (3)	0.013 (2)	0.006 (3)	−0.006 (3)
C5	0.081 (4)	0.060 (4)	0.052 (3)	0.025 (3)	0.031 (3)	0.009 (3)
C6	0.075 (4)	0.045 (3)	0.049 (3)	0.018 (3)	0.024 (3)	0.016 (3)
C7	0.045 (3)	0.025 (2)	0.032 (2)	0.003 (2)	−0.003 (2)	0.004 (2)
C8	0.064 (3)	0.038 (3)	0.037 (3)	0.011 (3)	0.003 (2)	0.002 (2)
C9	0.056 (3)	0.046 (3)	0.040 (3)	0.006 (3)	0.004 (2)	0.003 (2)
C10	0.060 (4)	0.073 (4)	0.069 (4)	−0.018 (3)	0.014 (3)	0.013 (3)
C11	0.068 (4)	0.069 (4)	0.046 (3)	0.010 (3)	−0.005 (3)	−0.001 (3)
C12	0.060 (4)	0.072 (4)	0.068 (4)	−0.012 (3)	−0.016 (3)	−0.005 (3)
C13	0.109 (6)	0.103 (6)	0.073 (5)	0.009 (5)	−0.016 (4)	0.032 (4)
C14	0.047 (3)	0.033 (3)	0.056 (3)	−0.012 (2)	−0.005 (3)	0.005 (3)
C15	0.032 (2)	0.027 (2)	0.033 (2)	−0.0045 (18)	0.0043 (19)	0.0029 (19)
C16	0.032 (2)	0.028 (2)	0.042 (3)	−0.0045 (18)	0.004 (2)	−0.003 (2)
C17	0.047 (3)	0.033 (3)	0.053 (3)	−0.009 (2)	0.000 (2)	−0.007 (2)
C18	0.068 (4)	0.055 (3)	0.040 (3)	−0.021 (3)	−0.015 (3)	0.001 (3)
C19	0.067 (4)	0.050 (3)	0.039 (3)	−0.021 (3)	−0.012 (3)	0.010 (2)
C20	0.051 (3)	0.030 (2)	0.031 (2)	−0.009 (2)	0.000 (2)	0.003 (2)
C21	0.076 (4)	0.042 (3)	0.034 (3)	−0.013 (3)	−0.008 (3)	0.004 (2)
C22	0.064 (3)	0.051 (3)	0.045 (3)	−0.006 (2)	−0.005 (2)	−0.001 (3)
C23	0.081 (3)	0.066 (4)	0.066 (4)	0.016 (3)	−0.012 (3)	0.015 (3)
C24	0.085 (4)	0.084 (5)	0.052 (3)	−0.014 (3)	0.006 (3)	0.001 (3)
C25	0.164 (8)	0.097 (6)	0.043 (3)	−0.030 (6)	−0.006 (4)	−0.004 (4)
C26	0.077 (4)	0.148 (8)	0.075 (5)	0.003 (4)	0.018 (3)	−0.010 (5)

### Geometric parameters (Å, °)

**Table:**

Mn1—O8	2.163 (3)	C8—C9	1.533 (7)
Mn1—N3	2.168 (4)	C9—C10	1.520 (7)
Mn1—N6	2.171 (4)	C9—C11	1.525 (7)
Mn1—O7	2.173 (3)	C10—H10A	0.9600
Mn1—N4	2.337 (4)	C10—H10B	0.9600
Mn1—N1	2.348 (4)	C10—H10C	0.9600
N1—C6	1.318 (6)	C11—C13	1.519 (8)
N1—C2	1.350 (5)	C11—C12	1.527 (8)
N2—C7	1.301 (5)	C11—H11	0.9800
N2—C9	1.475 (6)	C12—H12A	0.9600
N2—H2	0.8600	C12—H12B	0.9600
N3—C7	1.345 (5)	C12—H12C	0.9600
N3—C8	1.369 (6)	C13—H13A	0.9600
N4—C19	1.325 (6)	C13—H13B	0.9600
N4—C15	1.351 (5)	C13—H13C	0.9600
N5—C20	1.303 (6)	C14—C16	1.531 (6)
N5—C22	1.468 (7)	C15—C16	1.398 (6)
N5—H5	0.8600	C15—C20	1.489 (6)
N6—C20	1.340 (5)	C16—C17	1.390 (7)
N6—C21	1.377 (6)	C17—C18	1.363 (7)
O1—C1	1.231 (6)	C17—H17	0.9300
O2—C1	1.243 (6)	C18—C19	1.376 (7)
O3—C8	1.218 (6)	C18—H18	0.9300
O4—C14	1.222 (6)	C19—H19	0.9300
O5—C14	1.225 (6)	C21—C22	1.556 (7)
O6—C21	1.222 (6)	C22—C24	1.492 (8)
O7—H7A	0.8499	C22—C23	1.531 (8)
O7—H7B	0.8500	C23—H23A	0.9600
O8—H8A	0.8500	C23—H23B	0.9600
O8—H8B	0.8500	C23—H23C	0.9600
C1—C3	1.536 (6)	C24—C26	1.534 (10)
C2—C3	1.403 (6)	C24—C25	1.539 (9)
C2—C7	1.485 (6)	C24—H24	0.9800
C3—C4	1.393 (7)	C25—H25A	0.9600
C4—C5	1.372 (7)	C25—H25B	0.9600
C4—H4	0.9300	C25—H25C	0.9600
C5—C6	1.372 (7)	C26—H26A	0.9600
C5—H5A	0.9300	C26—H26B	0.9600
C6—H6	0.9300	C26—H26C	0.9600
			
O8—Mn1—N3	102.29 (13)	H10B—C10—H10C	109.5
O8—Mn1—N6	86.22 (13)	C13—C11—C9	112.7 (5)
N3—Mn1—N6	166.75 (15)	C13—C11—C12	111.7 (5)
O8—Mn1—O7	95.15 (13)	C9—C11—C12	112.4 (5)
N3—Mn1—O7	86.80 (13)	C13—C11—H11	106.5
N6—Mn1—O7	102.69 (14)	C9—C11—H11	106.5
O8—Mn1—N4	157.06 (13)	C12—C11—H11	106.5
N3—Mn1—N4	100.64 (14)	C11—C12—H12A	109.5
N6—Mn1—N4	71.08 (13)	C11—C12—H12B	109.5
O7—Mn1—N4	86.87 (13)	H12A—C12—H12B	109.5
O8—Mn1—N1	86.55 (13)	C11—C12—H12C	109.5
N3—Mn1—N1	71.07 (13)	H12A—C12—H12C	109.5
N6—Mn1—N1	99.70 (14)	H12B—C12—H12C	109.5
O7—Mn1—N1	157.60 (13)	C11—C13—H13A	109.5
N4—Mn1—N1	100.21 (14)	C11—C13—H13B	109.5
C6—N1—C2	119.1 (4)	H13A—C13—H13B	109.5
C6—N1—Mn1	122.9 (3)	C11—C13—H13C	109.5
C2—N1—Mn1	116.7 (3)	H13A—C13—H13C	109.5
C7—N2—C9	109.8 (4)	H13B—C13—H13C	109.5
C7—N2—H2	125.1	O4—C14—O5	124.1 (5)
C9—N2—H2	125.1	O4—C14—C16	114.9 (5)
C7—N3—C8	106.7 (4)	O5—C14—C16	121.1 (5)
C7—N3—Mn1	118.7 (3)	N4—C15—C16	122.3 (4)
C8—N3—Mn1	134.2 (3)	N4—C15—C20	110.3 (4)
C19—N4—C15	118.9 (4)	C16—C15—C20	127.4 (4)
C19—N4—Mn1	122.4 (3)	C17—C16—C15	116.3 (4)
C15—N4—Mn1	117.3 (3)	C17—C16—C14	115.3 (4)
C20—N5—C22	110.7 (4)	C15—C16—C14	128.4 (4)
C20—N5—H5	124.7	C18—C17—C16	121.5 (4)
C22—N5—H5	124.7	C18—C17—H17	119.2
C20—N6—C21	106.6 (4)	C16—C17—H17	119.2
C20—N6—Mn1	118.5 (3)	C17—C18—C19	118.2 (5)
C21—N6—Mn1	133.7 (3)	C17—C18—H18	120.9
Mn1—O7—H7A	111.9	C19—C18—H18	120.9
Mn1—O7—H7B	110.5	N4—C19—C18	122.7 (5)
H7A—O7—H7B	108.4	N4—C19—H19	118.6
Mn1—O8—H8A	111.1	C18—C19—H19	118.6
Mn1—O8—H8B	111.5	N5—C20—N6	114.9 (4)
H8A—O8—H8B	107.4	N5—C20—C15	125.4 (4)
O1—C1—O2	124.6 (5)	N6—C20—C15	119.6 (4)
O1—C1—C3	114.4 (4)	O6—C21—N6	125.0 (5)
O2—C1—C3	121.0 (4)	O6—C21—C22	125.7 (4)
N1—C2—C3	122.3 (4)	N6—C21—C22	109.1 (4)
N1—C2—C7	110.6 (4)	N5—C22—C24	109.6 (5)
C3—C2—C7	127.2 (4)	N5—C22—C23	112.0 (5)
C4—C3—C2	116.1 (4)	C24—C22—C23	112.2 (5)
C4—C3—C1	115.1 (4)	N5—C22—C21	98.3 (4)
C2—C3—C1	128.9 (4)	C24—C22—C21	113.7 (5)
C5—C4—C3	121.3 (4)	C23—C22—C21	110.4 (5)
C5—C4—H4	119.3	C22—C23—H23A	109.5
C3—C4—H4	119.3	C22—C23—H23B	109.5
C4—C5—C6	118.0 (5)	H23A—C23—H23B	109.5
C4—C5—H5A	121.0	C22—C23—H23C	109.5
C6—C5—H5A	121.0	H23A—C23—H23C	109.5
N1—C6—C5	123.1 (5)	H23B—C23—H23C	109.5
N1—C6—H6	118.5	C22—C24—C26	111.0 (5)
C5—C6—H6	118.5	C22—C24—C25	112.4 (6)
N2—C7—N3	114.9 (4)	C26—C24—C25	111.0 (6)
N2—C7—C2	125.4 (4)	C22—C24—H24	107.4
N3—C7—C2	119.7 (4)	C26—C24—H24	107.4
O3—C8—N3	126.1 (4)	C25—C24—H24	107.4
O3—C8—C9	124.4 (4)	C24—C25—H25A	109.5
N3—C8—C9	109.6 (4)	C24—C25—H25B	109.5
N2—C9—C10	112.1 (4)	H25A—C25—H25B	109.5
N2—C9—C11	110.0 (4)	C24—C25—H25C	109.5
C10—C9—C11	113.6 (5)	H25A—C25—H25C	109.5
N2—C9—C8	99.1 (4)	H25B—C25—H25C	109.5
C10—C9—C8	110.9 (5)	C24—C26—H26A	109.5
C11—C9—C8	110.3 (4)	C24—C26—H26B	109.5
C9—C10—H10A	109.5	H26A—C26—H26B	109.5
C9—C10—H10B	109.5	C24—C26—H26C	109.5
H10A—C10—H10B	109.5	H26A—C26—H26C	109.5
C9—C10—H10C	109.5	H26B—C26—H26C	109.5
H10A—C10—H10C	109.5		
			
O8—Mn1—N1—C6	−79.3 (4)	C3—C2—C7—N3	175.8 (4)
N3—Mn1—N1—C6	176.5 (5)	C7—N3—C8—O3	−179.3 (5)
N6—Mn1—N1—C6	6.3 (4)	Mn1—N3—C8—O3	9.4 (9)
O7—Mn1—N1—C6	−174.4 (4)	C7—N3—C8—C9	1.4 (6)
N4—Mn1—N1—C6	78.7 (4)	Mn1—N3—C8—C9	−169.9 (3)
O8—Mn1—N1—C2	87.5 (3)	C7—N2—C9—C10	−117.0 (5)
N3—Mn1—N1—C2	−16.8 (3)	C7—N2—C9—C11	115.7 (5)
N6—Mn1—N1—C2	173.1 (3)	C7—N2—C9—C8	0.1 (5)
O7—Mn1—N1—C2	−7.7 (6)	O3—C8—C9—N2	179.8 (5)
N4—Mn1—N1—C2	−114.6 (3)	N3—C8—C9—N2	−0.9 (5)
O8—Mn1—N3—C7	−68.3 (3)	O3—C8—C9—C10	−62.2 (7)
N6—Mn1—N3—C7	60.8 (8)	N3—C8—C9—C10	117.0 (5)
O7—Mn1—N3—C7	−162.9 (3)	O3—C8—C9—C11	64.5 (7)
N4—Mn1—N3—C7	110.9 (3)	N3—C8—C9—C11	−116.2 (5)
N1—Mn1—N3—C7	13.6 (3)	N2—C9—C11—C13	73.7 (6)
O8—Mn1—N3—C8	102.1 (5)	C10—C9—C11—C13	−52.8 (7)
N6—Mn1—N3—C8	−128.7 (6)	C8—C9—C11—C13	−178.1 (5)
O7—Mn1—N3—C8	7.5 (5)	N2—C9—C11—C12	−53.7 (6)
N4—Mn1—N3—C8	−78.7 (5)	C10—C9—C11—C12	179.8 (5)
N1—Mn1—N3—C8	−175.9 (5)	C8—C9—C11—C12	54.6 (6)
O8—Mn1—N4—C19	−173.7 (4)	C19—N4—C15—C16	2.4 (7)
N3—Mn1—N4—C19	8.3 (4)	Mn1—N4—C15—C16	−164.7 (3)
N6—Mn1—N4—C19	177.6 (4)	C19—N4—C15—C20	−178.9 (4)
O7—Mn1—N4—C19	−77.8 (4)	Mn1—N4—C15—C20	14.0 (5)
N1—Mn1—N4—C19	80.7 (4)	N4—C15—C16—C17	−2.0 (7)
O8—Mn1—N4—C15	−7.0 (6)	C20—C15—C16—C17	179.6 (4)
N3—Mn1—N4—C15	174.9 (3)	N4—C15—C16—C14	177.9 (4)
N6—Mn1—N4—C15	−15.8 (3)	C20—C15—C16—C14	−0.6 (8)
O7—Mn1—N4—C15	88.8 (3)	O4—C14—C16—C17	22.3 (6)
N1—Mn1—N4—C15	−112.6 (3)	O5—C14—C16—C17	−156.7 (6)
O8—Mn1—N6—C20	−162.1 (4)	O4—C14—C16—C15	−157.6 (5)
N3—Mn1—N6—C20	67.3 (7)	O5—C14—C16—C15	23.5 (8)
O7—Mn1—N6—C20	−67.6 (4)	C15—C16—C17—C18	−0.4 (7)
N4—Mn1—N6—C20	14.5 (3)	C14—C16—C17—C18	179.7 (5)
N1—Mn1—N6—C20	112.1 (4)	C16—C17—C18—C19	2.2 (8)
O8—Mn1—N6—C21	3.2 (5)	C15—N4—C19—C18	−0.5 (8)
N3—Mn1—N6—C21	−127.4 (6)	Mn1—N4—C19—C18	166.0 (4)
O7—Mn1—N6—C21	97.6 (5)	C17—C18—C19—N4	−1.8 (9)
N4—Mn1—N6—C21	179.8 (5)	C22—N5—C20—N6	−1.6 (7)
N1—Mn1—N6—C21	−82.7 (5)	C22—N5—C20—C15	177.6 (5)
C6—N1—C2—C3	3.1 (7)	C21—N6—C20—N5	−2.7 (6)
Mn1—N1—C2—C3	−164.1 (3)	Mn1—N6—C20—N5	166.2 (4)
C6—N1—C2—C7	−176.3 (4)	C21—N6—C20—C15	178.1 (4)
Mn1—N1—C2—C7	16.5 (5)	Mn1—N6—C20—C15	−13.0 (6)
N1—C2—C3—C4	−2.8 (6)	N4—C15—C20—N5	179.5 (5)
C7—C2—C3—C4	176.5 (4)	C16—C15—C20—N5	−1.9 (8)
N1—C2—C3—C1	177.9 (4)	N4—C15—C20—N6	−1.4 (6)
C7—C2—C3—C1	−2.8 (7)	C16—C15—C20—N6	177.2 (4)
O1—C1—C3—C4	22.3 (6)	C20—N6—C21—O6	−178.5 (6)
O2—C1—C3—C4	−156.6 (5)	Mn1—N6—C21—O6	15.0 (9)
O1—C1—C3—C2	−158.4 (5)	C20—N6—C21—C22	5.6 (6)
O2—C1—C3—C2	22.7 (7)	Mn1—N6—C21—C22	−160.9 (4)
C2—C3—C4—C5	−0.3 (7)	C20—N5—C22—C24	123.4 (6)
C1—C3—C4—C5	179.1 (5)	C20—N5—C22—C23	−111.5 (6)
C3—C4—C5—C6	2.9 (9)	C20—N5—C22—C21	4.5 (6)
C2—N1—C6—C5	−0.2 (9)	O6—C21—C22—N5	178.0 (6)
Mn1—N1—C6—C5	166.2 (5)	N6—C21—C22—N5	−6.1 (6)
C4—C5—C6—N1	−2.7 (10)	O6—C21—C22—C24	62.3 (8)
C9—N2—C7—N3	0.8 (6)	N6—C21—C22—C24	−121.8 (5)
C9—N2—C7—C2	−177.3 (4)	O6—C21—C22—C23	−64.8 (8)
C8—N3—C7—N2	−1.4 (6)	N6—C21—C22—C23	111.1 (5)
Mn1—N3—C7—N2	171.4 (3)	N5—C22—C24—C26	−56.6 (7)
C8—N3—C7—C2	176.8 (4)	C23—C22—C24—C26	178.3 (6)
Mn1—N3—C7—C2	−10.3 (5)	C21—C22—C24—C26	52.2 (7)
N1—C2—C7—N2	173.2 (5)	N5—C22—C24—C25	178.4 (5)
C3—C2—C7—N2	−6.2 (8)	C23—C22—C24—C25	53.4 (8)
N1—C2—C7—N3	−4.9 (6)	C21—C22—C24—C25	−72.8 (7)

### Hydrogen-bond geometry (Å, °)

**Table:**

*D*—H···*A*	*D*—H	H···*A*	*D*···*A*	*D*—H···*A*
N2—H2···O2	0.86	1.74	2.524 (5)	151
N5—H5···O5	0.86	1.76	2.535 (6)	149
O7—H7A···O3	0.85	2.09	2.838 (5)	147
O7—H7B···O1^i^	0.85	1.80	2.638 (5)	170
O8—H8A···O6	0.85	2.06	2.791 (5)	143
O8—H8B···O4^ii^	0.85	1.77	2.609 (5)	171

Symmetry codes: (i) −*x*+1/2, *y*−1/2, *z*; (ii) −*x*+1/2, *y*+1/2, *z*.
